# Wound Shock and Surgical Shock

**Published:** 1919-08-02

**Authors:** 


### The Hospital

The Workers' Newspaper of Administrative Medicine and Institutional Life, Administration, National Insurance and Health.

No. 1730, Vol. LXVI. SATURDAY, AUGUST 2, 1919. PRICE TWOPENCE

### WOUND SHOCK AND SURGICAL SHOCK.

In this year's Arris and Gale lectures before the Royal College of Surgeons of England, Mr. E. M. Cowell takes for his subject the relation between shock as seen on the battlefield, or in the military hospital, and shock as it occurs clinically in civilian surgery. He seeks to apply, very properly, the lessons learnt in war to the similar problems of peace; an aim which can only be described as both timely and desirable. The lectures do not, be it understood, contain the account of fresh research or of new discoveries: they are, as they are intended to be, a statement of the present position, a recapitulation of recent researches by various workers, rather than the account of any one individual's activities. As such, they deserve the careful study of medical practitioners, and more especially of operating surgeons. For hospital committeemen, who may be called upon by their medical staffs for various improved methods of combating and preventing shock during operations, or after accidents, the lectures are also worthy of attention, at least, in summary; and the same holds good to a limited extent for hospital architects and secretaries.

To start with, it may be said that the exact mechanism by which surgical shock is produced remains obscure. How and why the various factors which are now definitely established as contributory causes of shock bring about the train of symptoms characteristic of the developed condition, no one can as yet say for certain. Neither the original Crile theory, nor the opposing Yandell Henderson or "acapnia" theory, which for some years between them monopolised attention and aroused heated controversy between the respective partisans of these two eminent American researchers, is competent to fill the gap: and the same can be said of other less well known hypotheses. It is, nevertheless, possible that some day in the near future a satisfactory theory will be evolved, on which therapeutics may find a firm basis; at least a good many new facts, some of them apparently of great importance, have come to light as a consequence of war-time research. To this aspect of the Arris and Gale lectures it is hardly necessary to do more than refer here: what is of more importance is the practical upshot as it concerns prevention and treatment. In these respects there is definite and solid ground to tread. True, many of the recommendations for treatment are just those that were most relied on before the war, or are extensions of them; and much of the treatment is symptomatic, in the sense that it is directed to altering manifestations which may be causes or may be merely results of the state of surgical shock.

Mr. Cowell is distinctly, and we think rightly, sceptical as to the existence of shock unaccompanied by anatomical damage to one or other of the vital organs: of the purely psychical causation of shock, that is to say. He is prompt to admit that excitement, fatigue and strain in general may be amongst the pre-wound factors which predominate in the pathogenesis of wound shock. His first recommendation to the surgeon seeking to prevent shock is that he should remember the psychical aspect of the case and allay apprehension or excitement by quiet persuasion, suggestion, or the use of small doses of morphia. Next he counsels careful attention to the maintenance of the circulation and of the body heat. Should shock be already present in a patient on whom it is necessary to operate—as for instance, after a street or factory accident—the methods of preparation which were known in the casualty clearing stations as "resuscitation" are to be employed. These consist mainly in the application of warmth by the most suitable means to hand, while the patient is made as comfortable as possible and kept absolutely at rest. Various devices for converting the bed into a hot-air chamber were elaborated in France: these were usually ingenious makeshifts, and in civilian surgery it should be feasible to replace them by something a little more æsthetic. Next, fluids are to be freely supplied by the mouth or rectum, or gum saline may be given intravenously (not normal saline). If actual serious loss of blood has occurred, transfusion of whole blood from a suitable donor should be undertaken without delay. By these means a badly shocked patient can be got into condition to stand the necessary surgical operation by which the other causes of his shocked condition can be remedied. These latter are first and foremost sepsis and other sources of toxæmia, *e.g.*, damaged muscle tissue, gas gangrene, and so on. In this connection the surgeon and the anæsthetist are warned of the danger of chloroform, and to a less extent of ether, and are advised to rely on gas and oxygen anæsthesia, supplemented if necessary by regional anæsthesia. Finally, Mr. Cowell enunciates a theorem which may not command universal assent, though many keen observers had, we fancy, already adopted and acted on it before the war. "There is no evidence," he says, "of any permanent benefit following the injection of pituitrin, adsenalin, ergot preparations, atropine, caffeine, camphor, strychnine, or any of the numerous drugs recommended from time to time." At least, he will carry practising surgeons with him to the extent that these latter remedies are of far less importance than those previously enumerated.

